# Supplementary material for: Methylphenidate Analogues as a New Class of Potential Disease-Modifying Agents for Parkinson’s Disease: Evidence from Cell Models and Alpha-Synuclein Transgenic Mice
Source: Pharmaceutics. 2022 Jul 30;14(8):1595. doi: 10.3390/pharmaceutics14081595 (PMC9414221; doi:10.3390/pharmaceutics14081595)
Supplement: Supplementary file 1 [file pharmaceutics-14-01595-s001.zip › pharmaceutics-1752899-supplementary.pdf]

Supporting Information:

“Methylphenidate analogues as a new class of potential disease-modifying agents for Parkinson’s disease: evidence from cell models and alpha-synuclein transgenic mice”

Andrea Casiraghi[†], Francesca Longhena[†], Gaia Faustini, Giovanni Ribaudo, Lorenzo Suigo, Gisela Andrea Camacho-Hernandez, Federica Bono, Viviana Brembati, Amy Hauck Newman, Alessandra Gianoncelli, Valentina Straniero[*], Arianna Bellucci[‡] and Ermanno Valoti[‡]

Chemistry S-3
NMR spectra: S-10
threo 2-(piperidin-2-yl)-2-(p-tolyl) hydrochloride **(I-threo)** S-10
^{1}H NMR S-10
^{13}C NMR S-10
erythro N-Boc-methyl 2-(piperidin-2-yl)-2-(p-tolyl)acetate hydrochloride **(I-erythro)** S-11
^{1}H NMR S-11
^{13}C NMR S-11
threo methyl-2-(naphthalen-2-yl)-2-(piperidin-2-yl)acetate hydrochloride **(II-threo)** S-12
^{1}H NMR S-12
^{13}C NMR S-12
erythro methyl-2-([1,1'-biphenyl]-4-yl)-2-(piperidin-2-yl)acetate hydrochloride **(III-threo)** S-13
^{1}H NMR S-13
^{13}C NMR S-13
threo methyl 2-(1-methylpiperidin-2-yl)-2-(p-tolyl)acetate hydrochloride **(IV-threo)** S-14
^{1}H NMR S-14
^{13}C NMR S-14
HPLC S-15
HPLC method: S-15
HPLC sample preparation: S-15
I-*threo* S-16
I-*erythro* S-17
II-*threo* S-18
III-*threo* S-19
IV-*threo* S-20
Table S-1: S-21
Table S-2: S-21

Table S-3: S-22
Material and Methods Binding assay S-22
Figure S-1 .... S-24
Figure S-2 ....S-**Error! Bookmark not defined.**
Figure S-3 ....S-**Error! Bookmark not defined.**
Figure S-4 ....S-**Error! Bookmark not defined.**
Figure S-5 ....S-**Error! Bookmark not defined.**
Figure S-6 ....S-**Error! Bookmark not defined.**

Chemistry

N-Boc pipecolic acid[60] ***(1)**.* Pipecolic acid (10.0 g, 77.4 mmol) was dissolved in MeOH (50 mL), and TEA (11.9 mL, 85.2 mmol) was added. The mixture was heated up to 50 °C, and a solution of Boc_2O (33.8 g, 155 mmol) in MeOH (50 mL) was added. After stirring at RT for 20 hours, MeOH was evaporated under reduced pressure, and the residue was taken up in ethyl acetate (90 mL) and extracted with 10% $NaHCO_3$ (3x30 mL). The aqueous phase was acidified to pH=1 with 37% HCl, and the white precipitate was filtered and washed with water to afford 16.2 g (92%) of **1** as a white solid (mp: 130-133 °C). ^{1}H NMR (300 MHz, $CDCl_3$) δ 4.86 (bs, 1H), 4.14 – 3.81 (m, 1H), 3.06 – 2.79 (m, 1H), 2.29 – 2.10 (m, 1H), 1.75 – 1.11 (m, 14H).

N-Boc pipecolic acid morpholine amide [61] ***(2)**.* A suspension of **1** (7.80 g, 34.0 mmol), TBTU (14.20 g, 44.2 mmol) and TEA (6.64 mL, 47.6 mmol) in DCM (80 mL) was stirred, at RT, for 30 min. Morpholine (4.16 mL, 47.63 mmol) was added, and the mixture was stirred, at RT, for 24 hours. The organic phase was washed with 10% HCl (25 mL), 10% $NaHCO_3$ (25 mL) and saturated NaCl (25 mL), dried over Na_2SO_4 and evaporated under reduced pressure. Rotary evaporation was protracted to remove leftover tetramethyl urea and gave 8.58 g (85%) of **2** as a light orange waxy solid. ^{1}H NMR (300 MHz, $CDCl_3$) δ 5.00 – 4.56 (m, 1H), 3.90 (d, *J* = 12.4 Hz, 1H), 3.71 – 3.29 (m, 8H), 3.24 (t, *J* = 12.4 Hz, 1H), 1.90 – 1.49 (m, 5H), 1.38 (s, 10H).

N-Boc-piperidin-2-yl(p-tolyl)methanone [62] ***(3a)**.* An amount of 2.7M *n*-BuLi (10.4 mL, 28.2 mmol) was added dropwise to a solution of 4-bromotoluene (4.82 g, 28.2 mmol) in anhydrous THF (50 mL), at -78 °C, under nitrogen atmosphere. The mixture was stirred, at the same temperature, for 30 min, and a solution of **2** (2.80 g, 9.39 mmol) in anhydrous THF (30 mL) was added. After stirring for 4.5 hours, the reaction was quenched, at -78 °C, with a solution of 10% HCl (9 mL) in MeOH (20 mL). The mixture was brought to RT and ethyl acetate (50 mL) and phosphate buffer (50 mL) were added. THF was evaporated under reduced pressure, phases were separated, and the aqueous phase was extracted with ethyl acetate (2x25 mL). The collected organic phases were dried over Na_2SO_4 and evaporated under reduced pressure. The obtained crude was purified by column chromatography on silica gel. Elution with cyclohexane/ethyl acetate 95:5 gave 1.99 g (70%) of **3a** as a white solid (mp: 95-97 °C). ^{1}H NMR (300 MHz, $CDCl_3$) δ 7.81 (d, *J* = 7.7 Hz, 2H), 7.24 (d, *J* = 7.7 Hz, 2H), 5.54 (d, 1H), 3.93 (m, 1H), 3.16 (m, 1H), 2.40 (s, 3H), 2.20 – 1.95 (m, 1H), 1.91 – 1.71 (m, 1H), 1.72 – 1.23 (m, 13H).

*N-Boc-2-(1-(p-tolyl)vinyl)piperidine (**4a**).* Potassium tert-butylate (0.69 g, 6.18 mmol) was added to a solution of methyltriphenylphosphonium bromide (2.21 g, 6.18 mmol) in anhydrous THF (10 mL) under nitrogen atmosphere and the suspension was stirred for 10 min, at RT. A solution of **3a** in 15 mL of THF was added dropwise and the mixture was stirred, at RT, for 4 hours. The reaction was quenched with water (50 mL) and extracted with ethyl acetate (3x20 mL). The organic phase was dried over Na_2SO_4 and evaporated under reduced pressure, and the obtained crude was purified by filtration on silica gel plug (eluent cyclohexane/ethyl acetate 7:3) to give 0.97 g (90%) of **4a** as colorless oil. ^{1}H NMR (300 MHz, $CDCl_3$) δ 7.20 (d, *J* = 8.1 Hz, 2H), 7.11 (d, *J* = 8.1 Hz, 2H), 5.24 (m, 1H), 5.23 (s, 1H), 4.99 (s, 1H), 3.94 (d, *J* = 12.0 Hz, 1H), 2.89 (m, 1H), 2.33 (s, 3H), 1.80 – 1.67 (m, 1H), 1.62 – 1.44 (m, 3H), 1.45 – 1.26 (m, 11H).

*erythro and threo N-Boc-2-(piperidin-2-yl)-2-(p-tolyl)ethanol (**5a threo/erythro**).* An amount of 1M borane THF complex (22.9 mL, 22.9 mmol) was added dropwise to a solution of **4a** (3.45 g, 11.4 mmol) in anhydrous THF (50 mL) under nitrogen atmosphere. The mixture was stirred overnight, at RT, and water (20 mL), 2.5N NaOH (20 mL) and 35% H_2O_2 (40 mL) were added in sequence. Stirring was continued for additional 4 hours. Water (40 mL) was added, and the mixture was extracted with ethyl acetate (3x50 mL). The organic phase was washed with 5% sodium bisulfite (50 mL), dried over Na_2SO_4 and evaporated under reduced pressure. The obtained crude was purified by column chromatography on silica gel. Elution with cyclohexane/ethyl acetate 90:10 gave 1.54 g (42%) of **5a-*threo*** as a white solid (mp: 138-141 °C) and 0.12 g (22%) of **5a-*erythro*** as a colorless oil. ^{1}H NMR (300 MHz, $CDCl_3$) threo: δ 7.25 (d, *J* = 8.2 Hz, 2H), 7.12 (d, *J* = 8.2 Hz, 2H), 4.63 (m, 1H), 4.01 (m, 1H), 3.72 (m, 1H), 3.56 (m, 1H), 3.04 (m, 1H), 2.82 (dt, *J* = 13.2, 2.9 Hz, 1H), 2.32 (s, 3H), 1.65 – 1.17 (m, 15H); erythro: δ 7.13 (d, *J* = 8.2 Hz, 2H), 7.09 (d, *J* = 8.2 Hz, 2H), 4.47 (m, 1H), 3.82 (dd, *J* = 12.1, 5.9 Hz, 1H), 3.78 - 3.67 (m, 2H), 3.22 (m, 1H), 2.59 (m, 1H), 2.29 (s, 3H), 1.80 - 1.40 (m, 6H), 1.29 (s, 9H).

*threo N-Boc-2-(piperidin-2-yl)-2-(p-tolyl)acetic acid (**6a-threo**).* Pyridinium dichromate (0.62 g, 1.64 mmol) and 3Å molecular sieves (0.03 g) were added to a solution of **5a-*threo*** (0.15 g, 0.47 mmol) in DMF (2 mL), and the mixture was stirred overnight, at RT. Ethyl ether (5 mL) was added, and the reaction was quenched by adding 10% HCl (5 mL) dropwise, at 0 °C. The aqueous phase was extracted with ethyl ether (3x5 mL), and the collected organic phases were extracted with 10% NaOH (3x10 mL). The collected aqueous phase was acidified pH=1 with conc. HCl and extracted with DCM (3x15 mL). The collected organic phases were dried over anhydrous Na_2SO_4 and evaporated under reduced pressure to give 0.11 g (70%) of **6a-*threo*** as a white waxy solid. ^{1}H NMR (300 MHz,

$CDCl_3$) 7.37 (d, *J* = 11.0 Hz, 2H), 7.07 (d, *J* = 11.0 Hz, 2H), 4.83 (m, 1H), 4.20 (d, *J* = 11.8 Hz, 1H), 3.97 (m, 1H), 3.19 (m, 1H), 2.28 (s, 3H), 1.75 – 1.17 (m, 6H), 1.48 (s, 9H).

threo methyl 2-(piperidin-2-yl)-2-(p-tolyl)acetate hydrochloride [26] ***(I-threo).*** **6a-*threo*** (0.60 g, 1.72 mmol) was dissolved in excess 2N HCl/MeOH and stirred, at RT, for 1 hour. Solvents were evaporated under reduced pressure, and the resulting crude was crystallized from toluene/MeOH 85:15 to give 0.22 g (44%) of **I-*threo*** as a white solid (mp: 205 °C with decomposition). Retention Time (HPLC) = 8.85'; A% (HPLC) = 95.6%. ^{1}H NMR (300 MHz, CD_3OD) δ 7.22 (d, *J* = 8.0 Hz, 2H), 7.15 (d, *J* = 8.0 Hz, 2H), 3.78 (m, 2H), 3.71 (s, 3H), 3.42 (m, 1H), 3.09 (dt, J = 12.8, 3.2 Hz, 1H), 2.33 (s, 3H), 1.84 (m, 2H), 1.68 – 1.42 (m, 3H), 1.33 (m, 1H). ^{13}C NMR (75 MHz, CD_3OD) δ 172.0, 138.5, 130.6, 129.6, 127.9, 57.9, 53.6, 51.9, 45.2, 26.3, 22.0, 21.3, 19.7.

*erythro N-Boc-2-(piperidin-2-yl)-2-(p-tolyl)acetic acid (**6a-erythro**).* Pyridinium dichromate (3.38 g, 8.98 mmol) and 3Å molecular sieves (0.67 g) were added to a solution of **5a-*erythro*** (0.82 g, 2.56 mmol) in DMF (10 mL), and the mixture was stirred overnight, at RT. Ethyl ether (25 mL) was added, and the reaction was quenched by adding 10% HCl (25 mL) dropwise, at 0 °C. The aqueous phase was extracted with ethyl ether (3x25 mL), and the collected organic phases were extracted with 10% NaOH (3x50 mL). The collected aqueous phases were brought to pH=1 with conc. HCl and extracted with DCM (3x50 mL). The collected organic phases were dried over Na_2SO_4 and evaporated under reduced pressure to give 0.54 g (63%) of **6a-*erythro*** as a white waxy solid. ^{1}H NMR (300 MHz, $CDCl_3$) 7.26 (d, *J* = 9.3 Hz, 2H), 7.07 (d, *J* = 9.3 Hz, 2H), 4.86 (m, 1H), 4.07 (d, *J* = 10.5 Hz, 1H), 3.95 (m, 1H), 2.99 (m, 1H), 2.32 (s, 3H), 1.58 – 1.25 (m, 6H), 1.44 (s, 9H).

erythro methyl 2-(piperidin-2-yl)-2-(p-tolyl)acetate hydrochloride [26] (***I-erythro***). An amount of 2M trimethylsilyldiazomethane (1.08 mL, 2.16 mmol) was added to a solution of **6a-*erythro*** (0.48 g, 1.44 mmol) and trimethyl orthoformate (2 mL) in toluene/MeOH (9 mL + 9 mL) under nitrogen atmosphere. The mixture was stirred overnight, at RT, and solvents were evaporated under reduced pressure to give 0.47 g (94%) of **7a-*erythro*** as a green oil. The product was dissolved in excess 2N HCl/MeOH and stirred, at RT, for 1 hour. Solvents were evaporated under reduced pressure, and the resulting crude was crystallized from toluene to give 0.26 g (68%) of **I-*erythro*** as a white solid (mp: 190.64 °C). Retention Time (HPLC) = 7.83'; A% (HPLC) = 95.1%. ^{1}H NMR (300 MHz, DMSO-d_6) δ 9.65 (bs, 1H), 7.64 (bs, 1H), 7.28 (d, *J* = 7.9 Hz, 2H), 7.20 (d, *J* = 7.9 Hz, 2H), 4.05 (d, *J* = 9.8 Hz, 1H), 3.67-3.54 (m, 1H), 3.59 (s, 3H), 3.12 – 3.03 (m, 1H), 2.84 – 2.71(m, 1H), 2.29 (s, 3H), 1.90 – 1.35 (m, 6H). ^{13}C NMR (75 MHz, DMSO-d_6) δ 171.5, 138.3, 130.7, 130.2, 129.2, 57.6, 53.9, 52.8, 45.3, 27.3, 22.1, 22.0, 21.2.

*N-Boc-naphthalen-2-yl(piperidin-2-yl)methanone (**3b**).* An amount of 2.7M *n*-BuLi (0.89 mL, 2.41 mmol) was added dropwise to a solution of 2-bromonaphthyl (0.5 g, 2.41 mmol) in anhydrous THF (5 mL), at -78 °C, under nitrogen atmosphere. The mixture was stirred, at the same temperature, for 45 min, and a solution of **2** (0.24 g, 0.79 mmol) in anhydrous THF (2.5 mL) was added. After stirring for 5 hours, the reaction was quenched with a solution of MeOH (1 mL) in THF (5 mL). Solvents were evaporated under reduced pressure and the residue was taken up in ethyl acetate (20 mL) and washed with 10% NaCl (3x10 mL) to a neutral pH. The organic phase was dried over Na_2SO_4 and evaporated under reduced pressure. The obtained crude was purified by column chromatography on silica gel. Elution with cyclohexane/ethyl acetate 90:10 gave 0.19 g (70%) of **3b** as a colorless oil. ^{1}H NMR (300 MHz, $CDCl_3$) δ 8.45 (m, 1H), 8.00 – 7.82 (m, 4H), 7.57 (m, 2H), 5.72 (m, 1H), 3.95 (m, 1H), 3.13 (m, 1H), 2.17 (m, 1H), 1.86 (m, 1H), 1.69 – 1.39 (m, 4H), 1.42 (s, 9H).

*N-Boc-2-(1-(naphthalen-2-yl)vinyl)piperidine (**4b**).* Potassium tert-butylate (0.29 g, 2.56 mmol) was added to a solution of methyltriphenylphosphonium bromide (0.92 g, 2.56 mmol) in anhydrous THF (5 mL) under nitrogen atmosphere. The mixture was stirred, at RT, for 10 min and a solution of **3b** (0.29 g, 0.85 mmol) in anhydrous THF (5 mL) was added. After stirring, at RT, for 7 hours, the reaction was quenched with water (25 mL) and extracted with ethyl acetate (3x10 mL). The organic phase was dried over Na_2SO_4 and evaporated under reduced pressure. Triphenylphosphine oxide was precipitated from IPE, and the solvent was evaporated under reduced pressure to yield 0.21 g (73%) of **4b** as a light-yellow oil. ^{1}H NMR (300 MHz, $CDCl_3$) 7.85– 7.37 (m, 7H), 5.47 – 5.36 (m, 1H), 5.39 (s, 1H), 5.13 (s, 1H), 4.00 – 3.90 (m, 1H), 2.97 – 2.84 (m, 1H), 1.88 – 1.76 (m, 2H), 1.68 – 1.35 (m, 4H), 1.45 (s, 9H).

*erythro and threo N-Boc-2-(naphthalen-2-yl)-2-(piperidin-2-yl)ethanol (**5b threo/erythro**).* An amount of 1M borane THF complex (0.99 mL, 0.99 mmol) was added dropwise to a solution of **4b** (0.15 g, 0.50 mmol) in anhydrous THF (2.5 mL) under nitrogen atmosphere. The mixture was stirred, at RT, for 6 hours, and water (1 mL), 2.5N NaOH (1 mL) and 35% H_2O_2 (2 mL) were added in succession. Stirring was continued over a weekend. Water (10 mL) was added, and the mixture was extracted with ethyl acetate (3x10 mL). The organic phase was dried over Na_2SO_4 and evaporated under reduced pressure. The obtained crude was purified by column chromatography on silica gel. Elution with cyclohexane/ethyl acetate 80:20 gave 0.06 g (34%) of **5b-*threo*** and 0.03 g (17%) of **5b-*erythro*** as colorless oils. ^{1}H NMR (300 MHz, $CDCl_3$) threo: δ 7.89 - 7.68 (m, 4H), 7.57 - 7.37 (m, 3H), 4.84 - 4.71 (m, 1H), 4.13 - 4.00 (m, 1H), 3.90 - 3.78 (m, 1H), 3.70 - 3.61 (m, 1H), 3.31 - 3.20 (m, 1H),

2.93 - 2.79 (m, 2H), 1.73 - 1.24 (m, 6H), 1.50 (s, 9H); erythro: δ 7.85 - 7.75 (m, 3H), 7.69 (s, 1H), 7.49 - 7.39 (m, 3H), 4.69 - 4.57 (m, 1H), 3.95 - 3.6 (m, 3H), 3.49 - 3.38 (m, 2H), 1.85 - 1.13 (m, 6H), 1.22 (s, 9H).

*threo N-Boc-2-(naphthalen-2-yl)-2-(piperidin-2-yl)acetic acid (**6b-threo**).* Pyridinium dichromate (0.48 g, 1.28 mmol) and 3Å molecular sieves (0.03 g) were added to a solution of **5b-*threo*** (0.13 g, 0.36 mmol) in DMF (3 mL) and the mixture was stirred overnight, at RT. Diethyl ether (5 mL) was added, and the reaction was quenched by adding 10% HCl (2.5 mL) dropwise, at 0 °C. The aqueous phase was extracted with ethyl ether (3x5 mL), and the collected organic phases were extracted with 10% NaOH (3x10 mL). The collected aqueous phases were brought to pH=1 with conc. HCl and extracted with DCM (3x15 mL). The collected organic phases were dried over Na_2SO_4 and evaporated under reduced pressure to give 0.10 g (75%) of **6b-*threo*** as a viscous green oil. The obtained crude was used in the next step without further purification.

threo methyl-2-(naphthalen-2-yl)-2-(piperidin-2-yl)acetate hydrochloride [26] *(**II-threo**).* An amount of **6b-*threo*** (51 mg, 0.13 mmol) was dissolved in excess 2N HCl/MeOH and stirred, at RT, for 1 hour. Solvents were evaporated under reduced pressure, and the resulting crude was decanted in IPE to give 10 mg (24%) of **II-*threo*** as a beige solid (mp: 205 °C with decomposition). Retention Time (HPLC) = 8.85'; A% (HPLC) = 95.6%. ^{1}H NMR (300 MHz, CD_3OD) δ 7.92 (d, *J* = 8.7 Hz, 1H), 7.87 (m, 1H), 7.80 (s, 1H), 7.53 (m, 2H), 7.39 (dd, *J* = 8.5, 1.7 Hz, 1H), 4.03 (d, J = 9.7 Hz, 1H), 3.95 (m, 1H), 3.74 (s, 3H), 3.45 (m, 1H), 3.13 (dt, *J* = 12.7, 3.3 Hz, 1H), 1.97 – 1.27 (m, 6H). ^{13}C NMR (75 MHz, CD_3OD) δ 171.8, 133.5, 133.2, 130.9, 128.9, 127.8, 127.5, 127.3, 126.5, 126.4, 124.9, 57.8, 54.0 52.0, 45.3, 26.5, 22.0, 21.3.

*N-Boc-[1,1'-biphenyl]-4-yl(piperidin-2-yl)methanone (**3c**).* An amount of 2.7M n-BuLi (3.72 mL, 10.06 mmol) was added dropwise to a solution of 4-bromobiphenyl (2.34 g, 10.06 mmol) in anhydrous THF (25 mL), at -78 °C, under nitrogen atmosphere. The mixture was stirred, at the same temperature, for 1 hour, and a solution of **2** (1.00 g, 3.35 mmol) in anhydrous THF (10 mL) was added. After stirring for 4 hours, the reaction was quenched with a solution of MeOH (1 mL) in THF (5 mL). Solvents were evaporated under reduced pressure, and the residue was taken up in ethyl acetate (30 mL) and washed with phosphate buffer (2x15 mL). The organic phase was dried over Na_2SO_4 and evaporated under reduced pressure. The obtained crude was purified by column chromatography on silica gel. Elution with cyclohexane/ethyl acetate 95:5 gave 0.53 g (48%) of **3c** as a colorless oil. ^{1}H NMR (300 MHz, $CDCl_3$) δ 7.93 (d, *J* = 6.5 Hz, 2H), 7.68 – 752 (m, 4H), 7.46 – 7.29

(m, 3H), 5.73 – 5.46 (m, 1H), 4.17 – 3.71 (m, 1H), 3.28 – 3.05 (m, 1H), 2.26 – 2.03 (m, 1H), 1.94 – 1.28 (m, 14H).

*N-Boc-2-(1-([1,1'-biphenyl]-4-yl)vinyl)piperidine (**4c**).* Potassium tert-butylate (0.27 g, 2.38 mmol) was added to a solution of methyltriphenylphosphonium bromide (0.85 g, 2.38 mmol) in anhydrous THF (3 mL) under nitrogen atmosphere. The mixture was stirred, at RT, for 10 min, and a solution of **3c** (0.58 g, 1.59 mmol) in anhydrous THF (7 mL) was added. After stirring overnight, at RT, the reaction was quenched with water (25 mL) and extracted with ethyl acetate (3x10 mL). The organic phase was dried over Na_2SO_4 and evaporated under reduced pressure, and the obtained crude was purified by filtration on silica gel plug (eluent cyclohexane/ethyl acetate 7:3) to give 0.56 g (96%) of **4c** as a white solid (mp: 119-124 °C). ^1H NMR (300 MHz, $CDCl_3$) 7.61 – 7.52 (m, 4H), 7.47 – 7.33 (m, 5H), 5.34 (m, 1H), 5.33 (s, 1H), 5.07 (s, 1H), 3.96 (m, 1H), 2.93 (m, 1H), 1.86 (m, 1H), 1.69 – 1.41 (m, 14H).

*erythro and threo N-Boc-2-([1,1'-biphenyl]-4-yl)-2-(piperidin-2-yl)ethanol (**5c threo/erythro**).* An amount of 1M borane THF complex (6.44 mL, 6.44 mmol) was added dropwise to a solution of **4c** (1.17 g, 3.22 mmol) in anhydrous THF (20 mL) under nitrogen atmosphere. The mixture was stirred overnight, at RT, and water (12 mL), 2.5N NaOH (12 mL) and 35% H_2O_2 (24 mL) were added in sequence. Stirring was continued for additional 2.5 hours. Water (25 mL) was added, and the mixture was extracted with ethyl acetate (3x30 mL). The organic phase was dried over Na_2SO_4 and evaporated under reduced pressure. The obtained crude was purified by column chromatography on silica gel. Elution with cyclohexane/ethyl acetate 75:25 gave 0.38 g (31%) of **5c-*threo*** as a white solid (mp: 150-152.5 °C) and 0.12 g (10%) of **5c-*erythro*** as a colorless oil. ^1H NMR (300 MHz, $CDCl_3$) threo: 7.63 – 7.51 (m, 4H), 7.68 – 7.28 (m, 5H), 4.69 (m, 1H), 4.07 (m, 1H), 3.76 (m, 1H), 3.60 (m, 1H), 3.16 (m, 1H), 2.87 (dt, J = 13.2, 2.4 Hz, 1H), 1.71 – 1.37 (m, 15H); erythro: δ 7.58 – 7.48 (m, 4H), 7.46 – 7.38 (m, 2H), 7.37 – 7.31 (m, 3H), 4.49 (m, 1H), 3.78 (m, 3H), 3.25 (m, 1H), 2.58 (m, 1H), 1.89 – 1.37 (m, 6H), 1.28 (s, 9H).

*threo N-Boc-2-([1,1'-biphenyl]-4-yl)-2-(piperidin-2-yl)acetic acid (**6c-threo**).* Pyridinium dichromate (1.31 g, 3.48 mmol) and 3Å molecular sieves (0.08 g) were added to a solution of **5c**-***threo*** (0.38 g, 0.99 mmol) in DMF (5 mL), and the mixture was stirred overnight, at RT. Diethyl ether (5 mL) was added, and the reaction was quenched by adding 10% HCl (5 mL) dropwise, at 0 °C. The aqueous phase was extracted with ethyl ether (3x5 mL), and the collected organic phases were extracted with 10% $NaHCO_3$ (3x10 mL). The collected aqueous phases were brought to pH=1 with conc. HCl

and extracted with DCM (3x15 mL). The collected organic phases were dried over Na_2SO_4 and evaporated under reduced pressure to give 0.33 g (84%) of **6c-*threo*** as a brown oil. ^{1}H NMR (300 MHz, $CDCl_3$) 7.62 – 7.28 (m, 9H), 5.11 – 4.77 (m, 1H), 4.17 (d, *J* = 11.7 Hz, 1H), 4.07 – 3.86 (m, 1H), 3.12 – 2.93 (m, 1H), 1.76 – 1.01 (m, 15H).

erythro methyl-2-([1,1'-biphenyl]-4-yl)-2-(piperidin-2-yl)acetate hydrochloride [26] ***(III-threo)***. An amount of 2M trimethylsilyldiazomethane (0.23 mL, 0.46 mmol) was added to a solution of **6c-*erythro*** (0.14 g, 0.35 mmol) in toluene/MeOH (3 mL + 3 mL) under nitrogen atmosphere. The mixture was stirred overnight, at RT, and solvents were evaporated under reduced pressure. The resulting crude was purified by column chromatography on silica gel. Elution with cyclohexane/ethyl acetate 90:10 gave 0.12 g (86%) of **7c-*threo*** as a colorless oil. The obtained product was dissolved in excess 2N HCl/MeOH and stirred, at RT, for 30 min. Solvents were evaporated under reduced pressure, and the resulting crude was slurried in hot toluene to give 30.4 mg (30%) of **III-*threo*** as a white solid (mp: 207-209 °C). Retention Time (HPLC) = 11.32'; A% (HPLC) = 98.6%. ^{1}H 0MR (300 MHz, CD_3OD) δ 7.68 – 7.58 (m, 4H), 7.47 – 7.31 (m, 5H), 3.97 – 3.80 (m, 2H), 3.75 (s, 3H), 3.46 (m, 1H), 3.12 (dt, *J* = 12.7, 3.1 Hz, 1H), 1.96 – 1.77 (m, 2H), 1.77 – 1.35 (m, 4H). ^{13}C NMR (75 MHz, CD_3OD) δ 171.8, 141.5, 140.0, 132.6, 128.7, 128.6, 127.5, 127.4, 126.5, 57.8, 53.6, 52.0, 45.3, 26.4, 22.0, 21.4.

threo methyl 2-(1-methylpiperidin-2-yl)-2-(p-tolyl)acetate hydrochloride [63] ***(IV-threo)***. An amount of **I-*threo*** (260 mg, 0.91 mmol) was dissolved in MeOH (20 mL), 37% paraformaldehyde (0.09 mL, 1.18 mmol) was added, and the mixture was stirred, at RT, for 20 min. Acetic acid (0.03 mL) and Pd/C 5% (KF=50%) (90 mg) were added, and the mixture was vigorously shaken under 2.5 atm hydrogen at RT for 2.5 hours. After filtering on a celite pad, solvent was evaporated under reduced pressure, and the residue was taken up in ethyl acetate (20 mL) and washed with 10% $NaHCO_3$ (10 mL) The aqueous phase was extracted with ethyl acetate (2x5mL), and the organic phases were collected, dried over Na_2SO_4 and evaporated under reduced pressure. The residue was treated with excess HCl/MeOH, and solvents were evaporated under reduced pressure. The resulting crude was treated with acetone (1 mL) to precipitate 47 mg (17%) **IV-*threo*** as a white solid (mp: 187 °C with decomposition). Retention Time (HPLC) = 8.97'; A% (HPLC) = 96.7%. ^{1}H NMR (300 MHz, CD_3OD, as freebase) δ 7.17 (d, *J* = 8.3 Hz, 2H), 7.12 (d, *J* = 8.3 Hz, 2H), 3.74 (d, *J* = 9.9 Hz, 1H), 3.63 (s, 3H), 3.15 (m, 1H), 2.87 (dt, *J* = 9.1, 4.2 Hz, 1H), 2.52 (m, 1H), 2.38 (s, 3H), 2.30 (s, 3H), 1.54 (m, 3H), 1.24 (m, 2H), 1.01 (m, 1H). ^{13}C NMR (75 MHz, $CDCl_3$, as freebase) δ 174.5, 137.0, 133.8, 129.3, 128.7, 63.4, 54.1, 54.0, 52.0, 40.8, 24.7, 23.0, 22.3, 21.0.

NMR spectra:

threo 2-(piperidin-2-yl)-2-(p-tolyl) hydrochloride ***(I-threo)***

^{1}H NMR

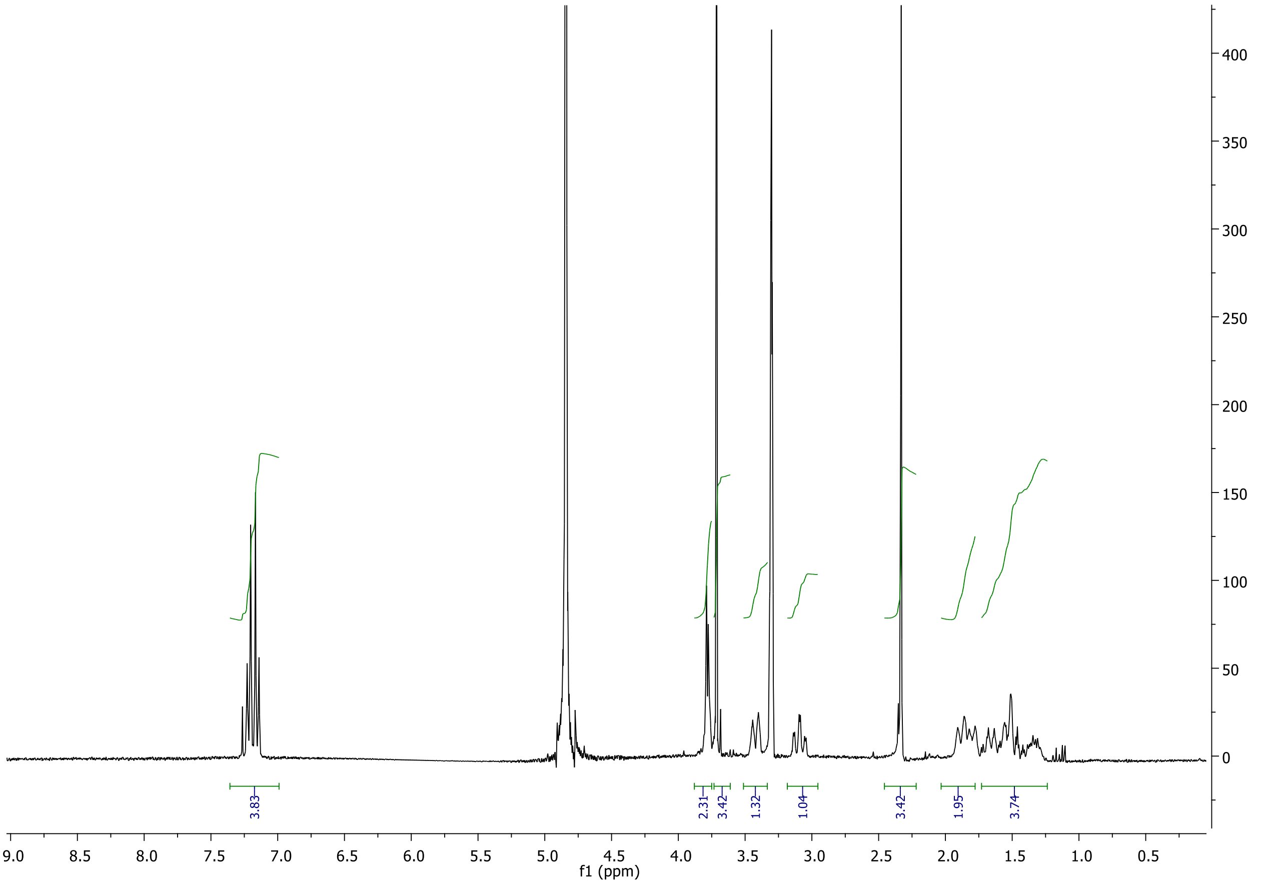

^{13}C NMR

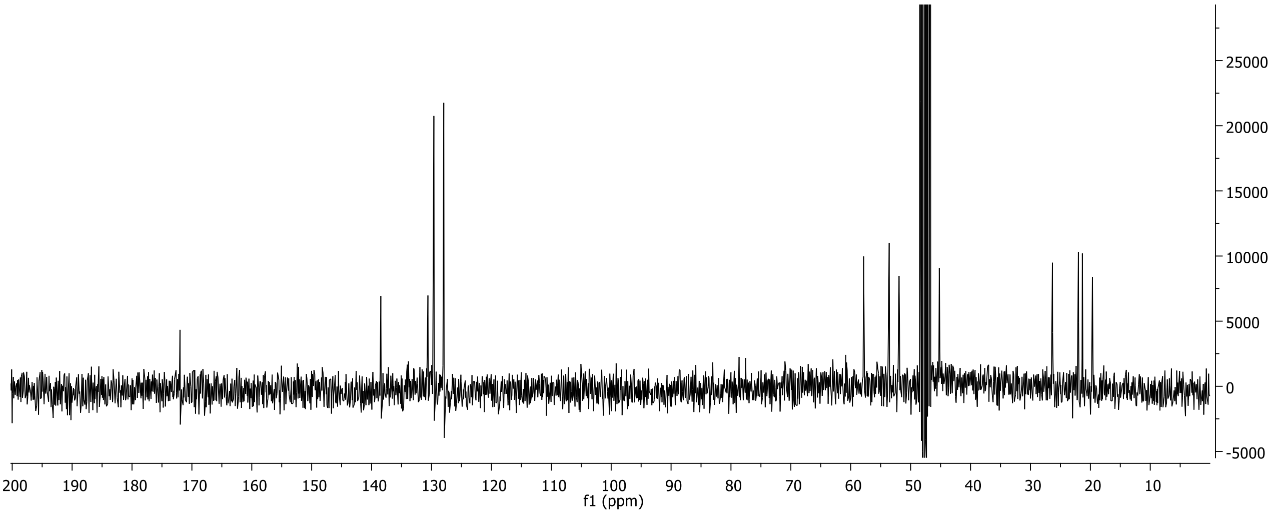

erythro N-Boc-methyl 2-(piperidin-2-yl)-2-(p-tolyl)acetate hydrochloride ***(I-erythro)***

^{1}H NMR

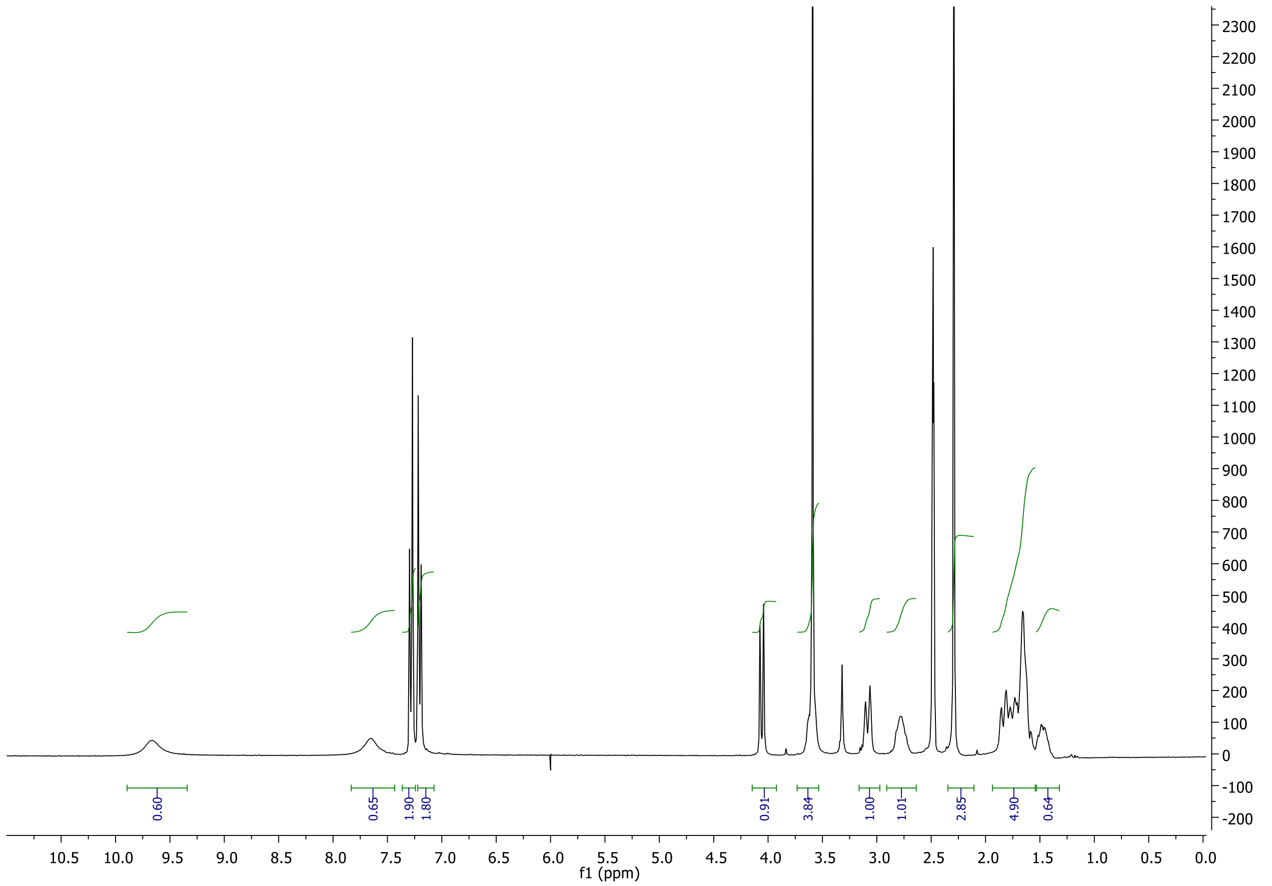

^{13}C NMR

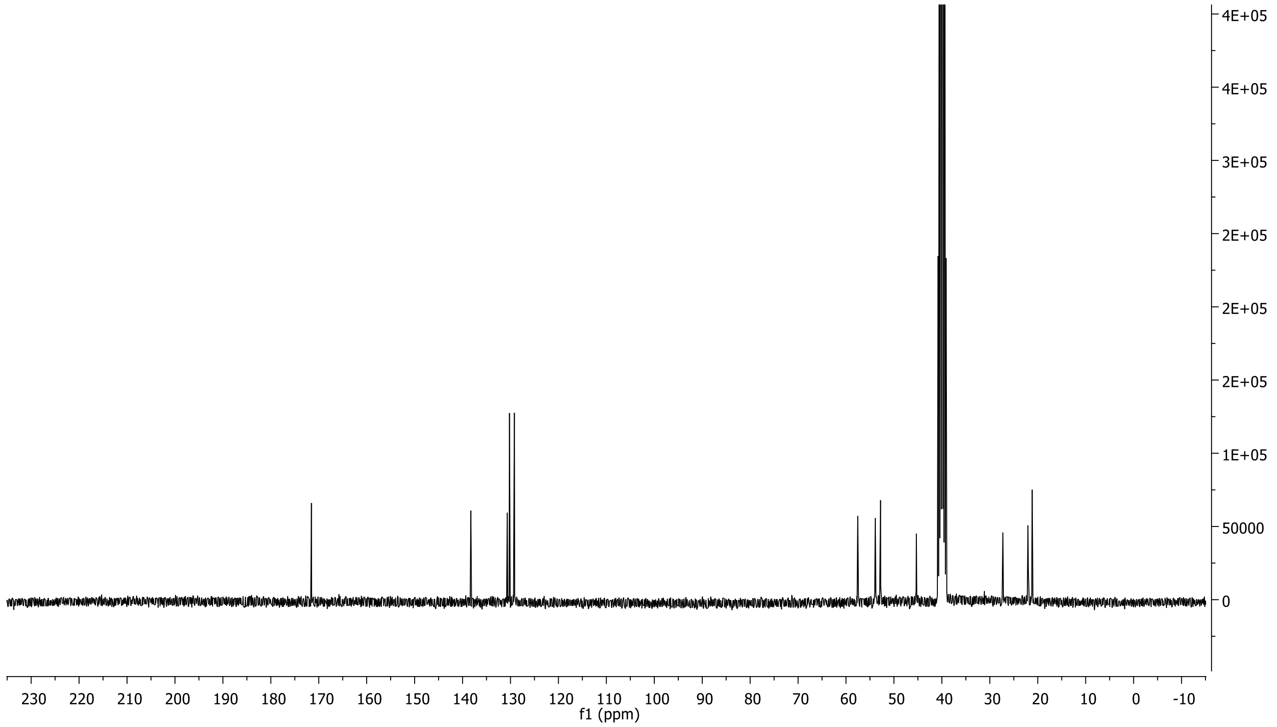

threo methyl-2-(naphthalen-2-yl)-2-(piperidin-2-yl)acetate hydrochloride ***(II-threo)***

^{1}H NMR

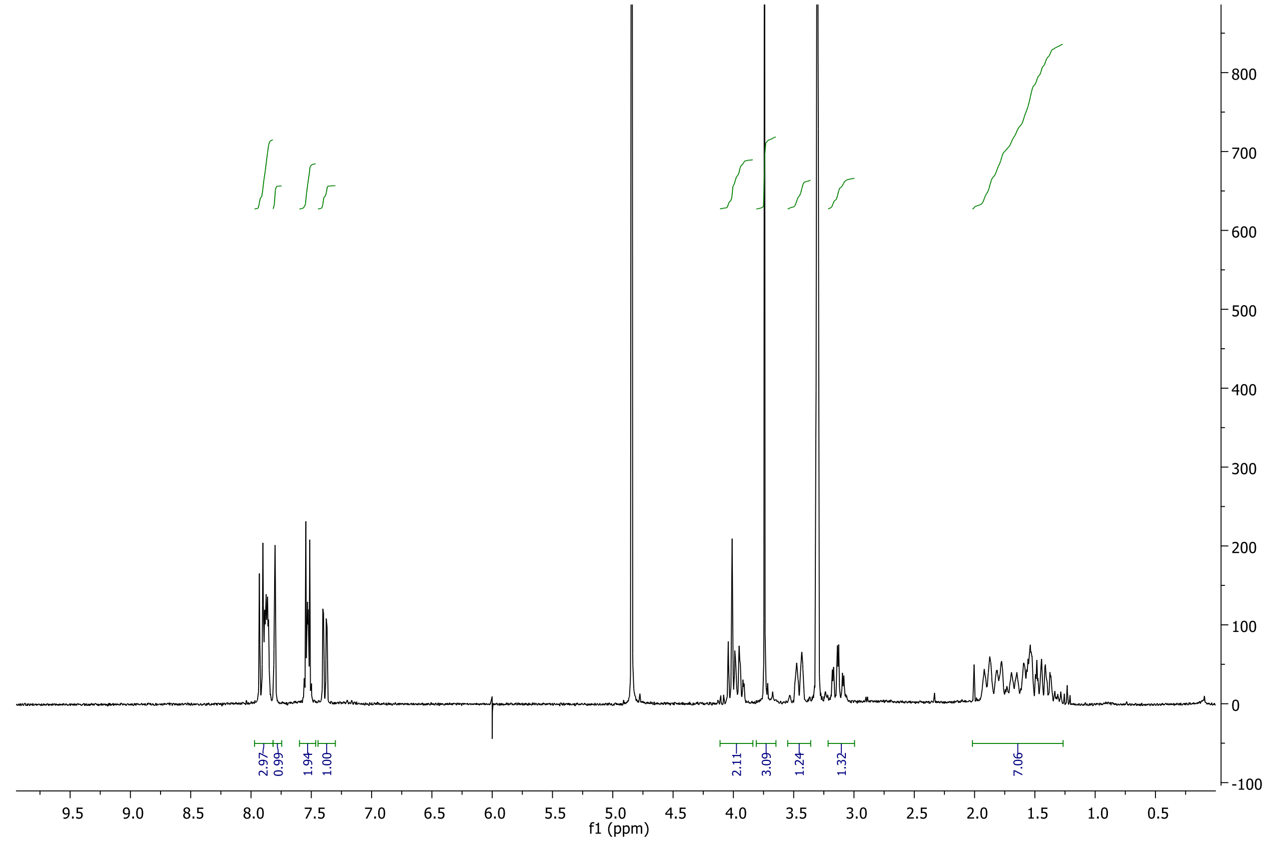

^{13}C NMR

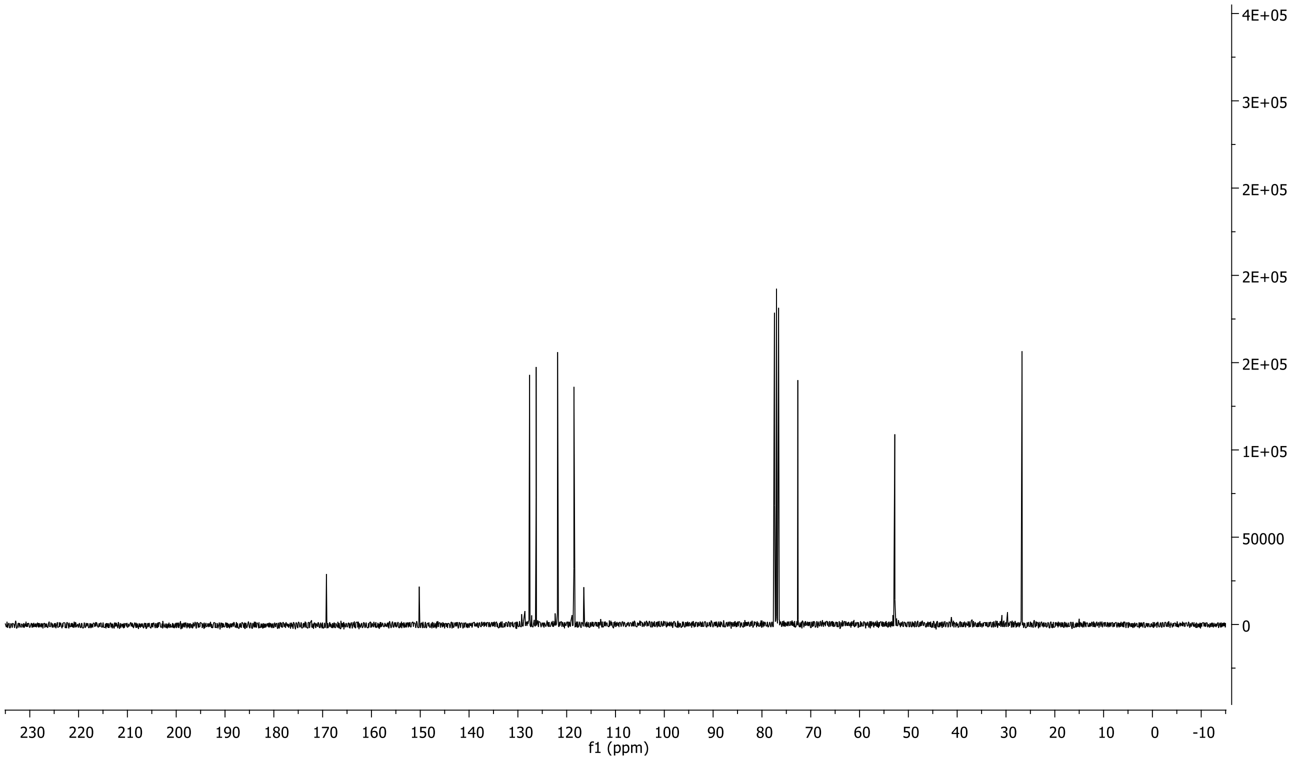

erythro methyl-2-([1,1’-biphenyl]-4-yl)-2-(piperidin-2-yl)acetate hydrochloride ***(III-threo)***

^{1}H NMR

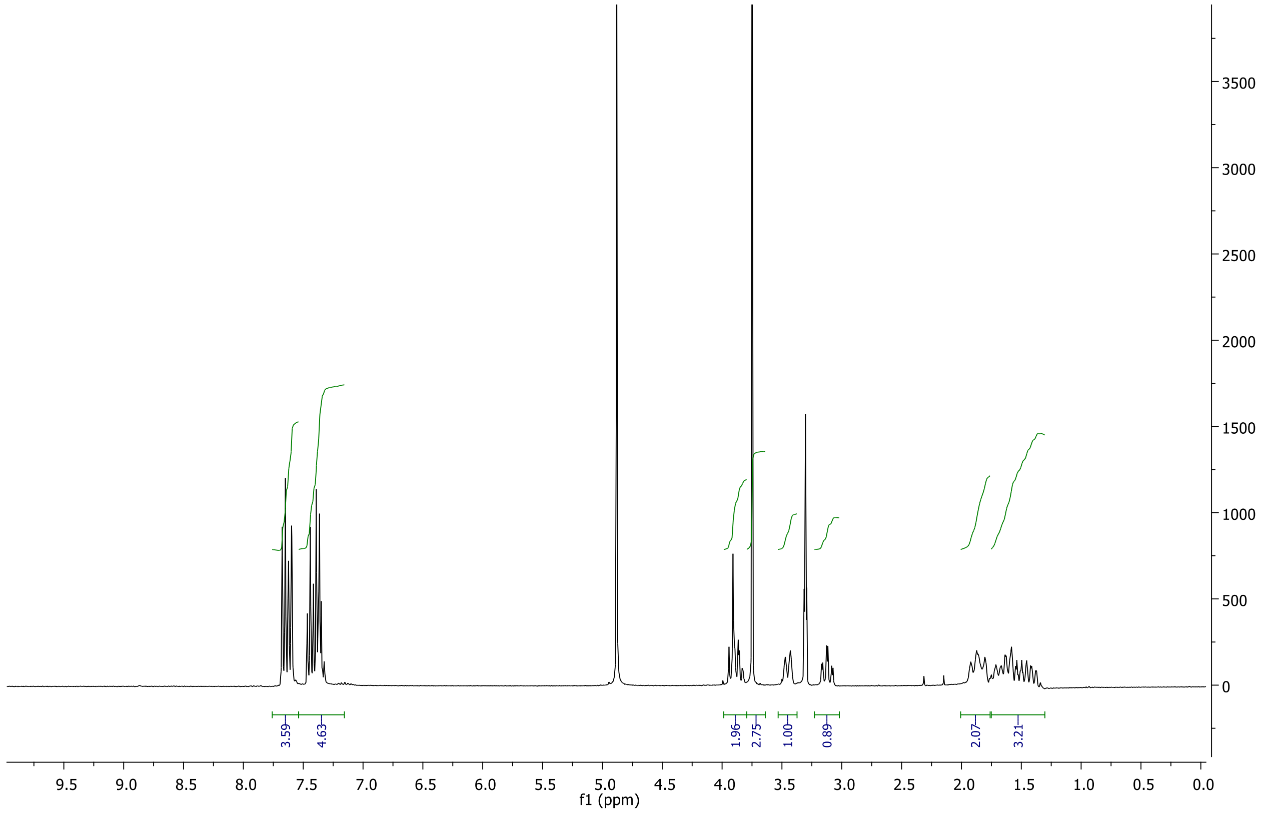

^{13}C NMR

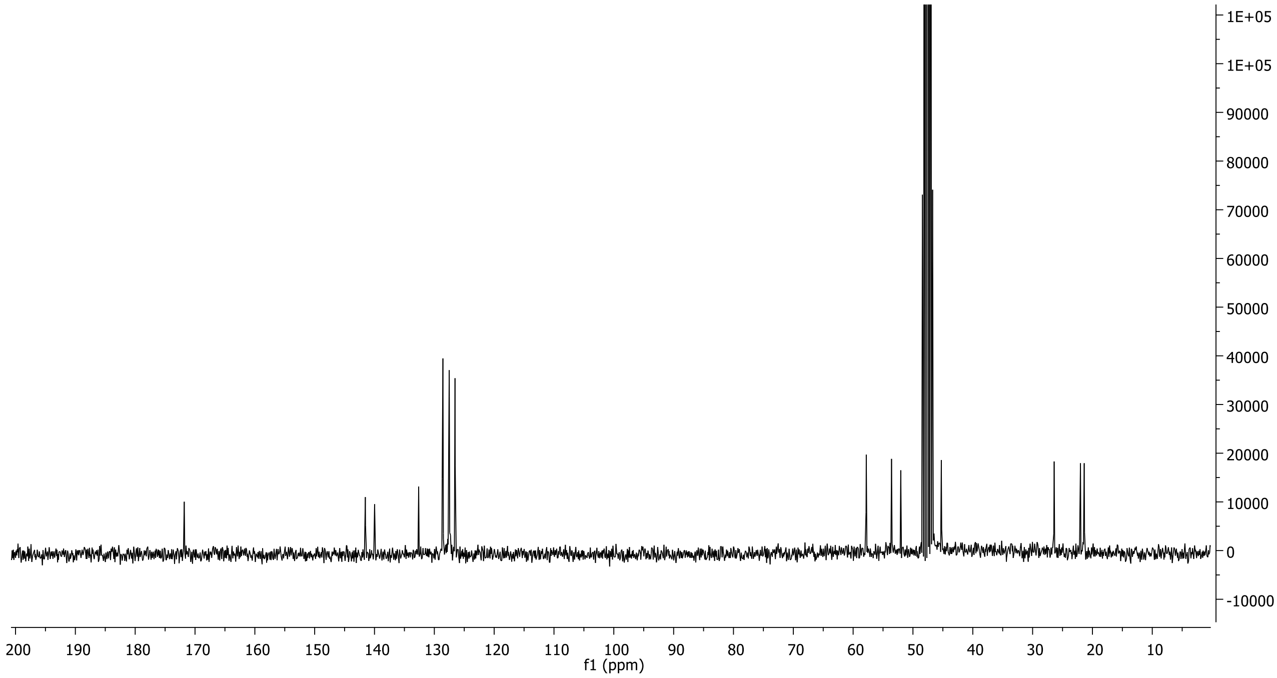

threo methyl 2-(1-methylpiperidin-2-yl)-2-(p-tolyl)acetate hydrochloride ***(IV-threo)***

^{1}H NMR

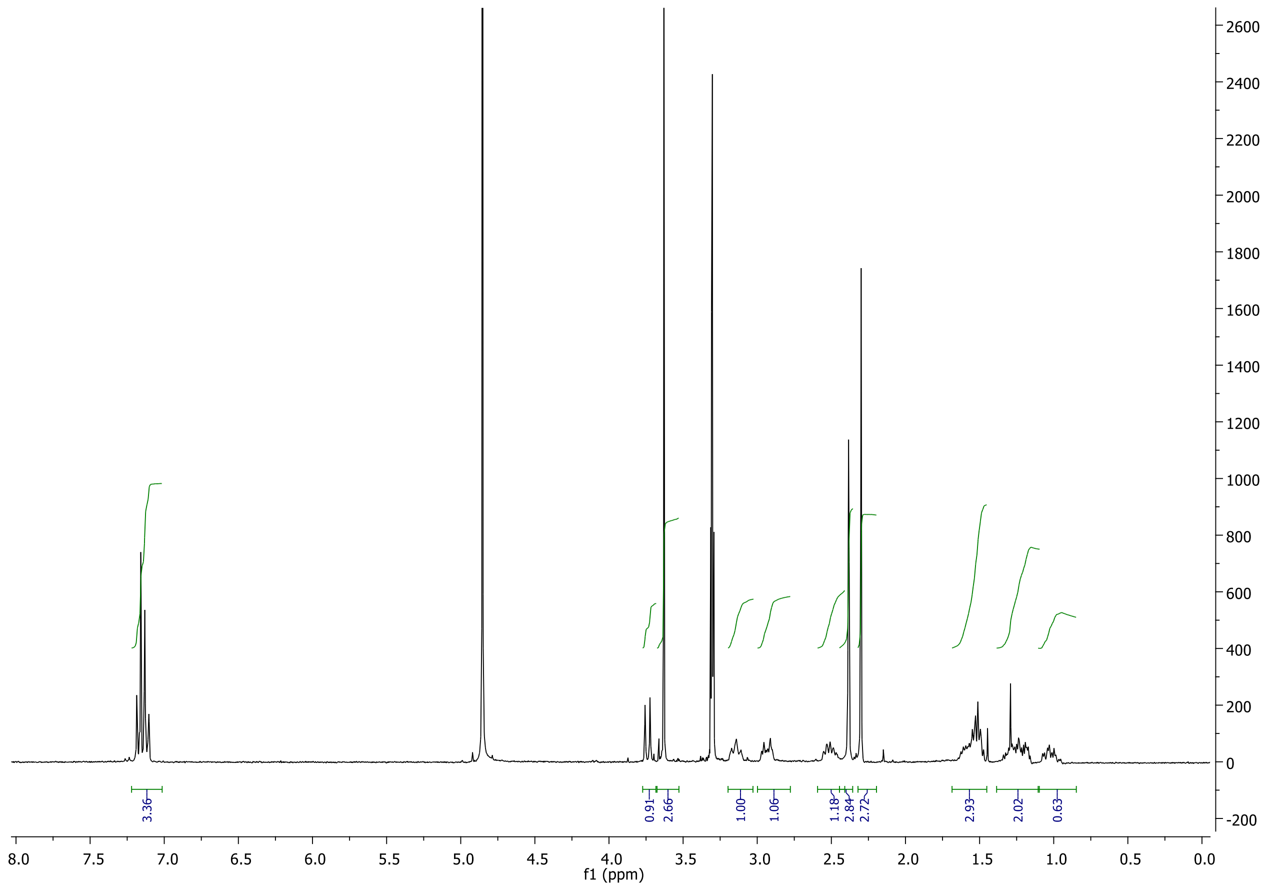

^{13}C NMR

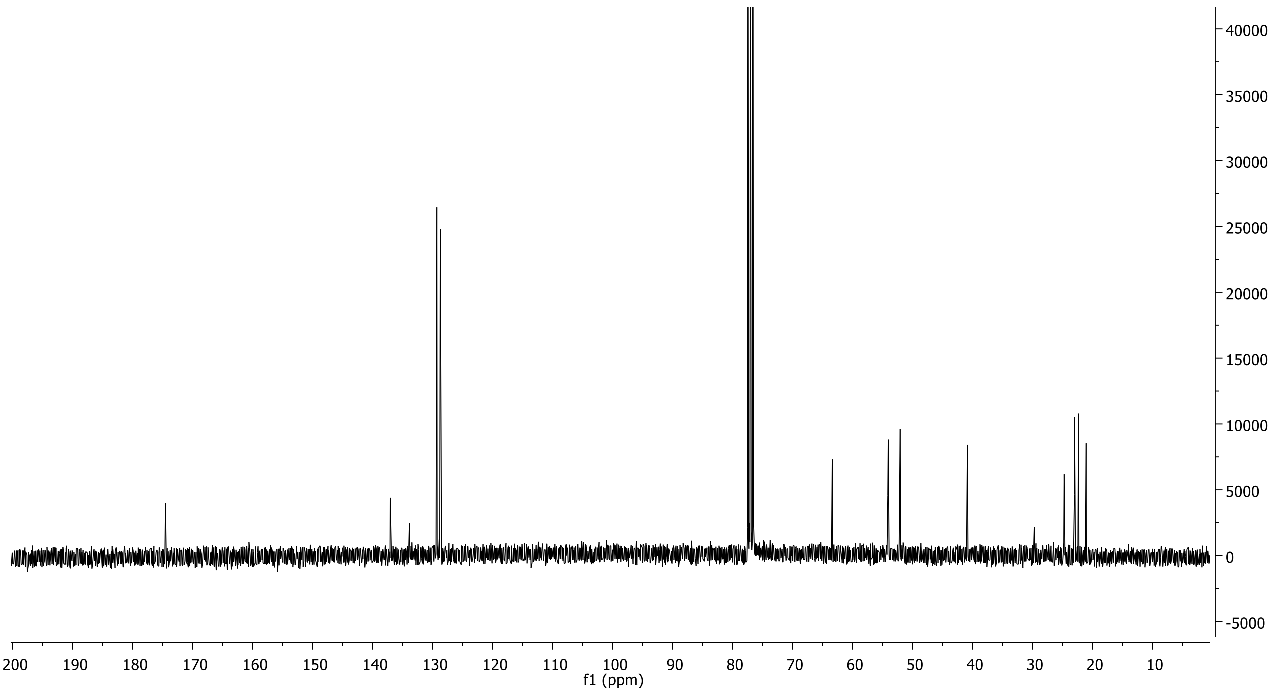

HPLC

HPLC method:

Time (minutes)	Solvents %		Flow rate (mL/min)
	Water +TFA 1‰	ACN + TFA‰	
0	90%	10%	1
20	10%	90%	1
25	10%	90%	1
30	90%	10%	1
35	90%	10%	1

HPLC sample preparation:

The HPLC method was set up using a Water Xbridge™ C-18 column flushed with freshly prepared 90% Water/10% I + TFA 1‰ until column pressure was stable. All the investigated samples were prepared through dissolution of the purified products in the selected mobile phase, at the approximate concentrations of 1 mg/mL, filtered through a 0.45 µm filter and analyzed. The injection volume was 20 µL. The purity was evaluated on each HPLC chromatogram, by recording the data at each absorption maximum.

l-*threo*

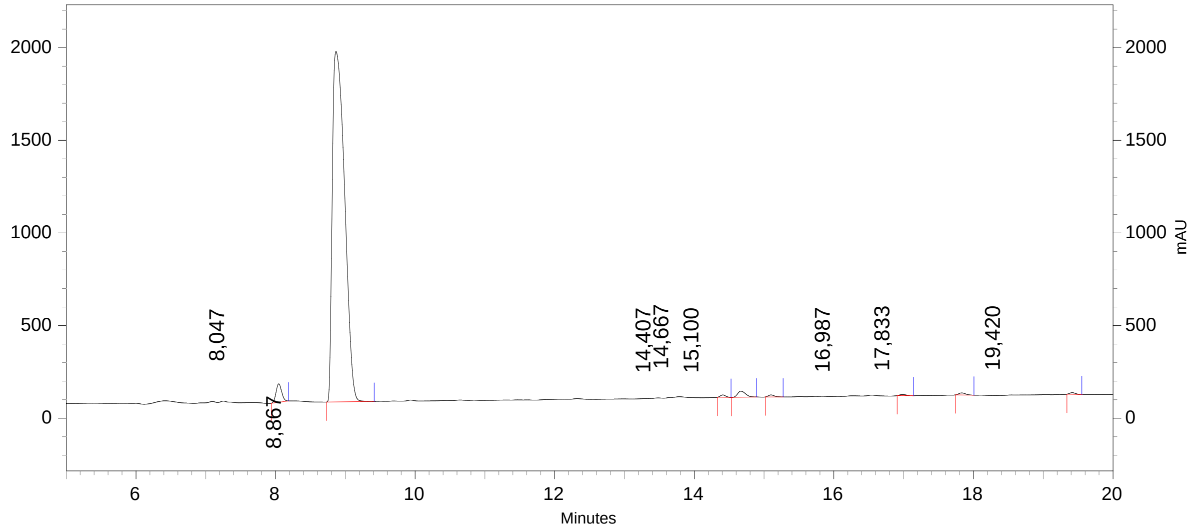

1: 217 nm, 4 nm Results

Retention Time	Area	Area %
8,047	2229699	2,29
8,867	92908378	95,44
14,407	271863	0,28
14,667	1035933	1,06
15,100	256245	0,26
16,987	147549	0,15
17,833	288463	0,30
19,420	210945	0,22
Totals	97349075	100,00

l-*erythro*

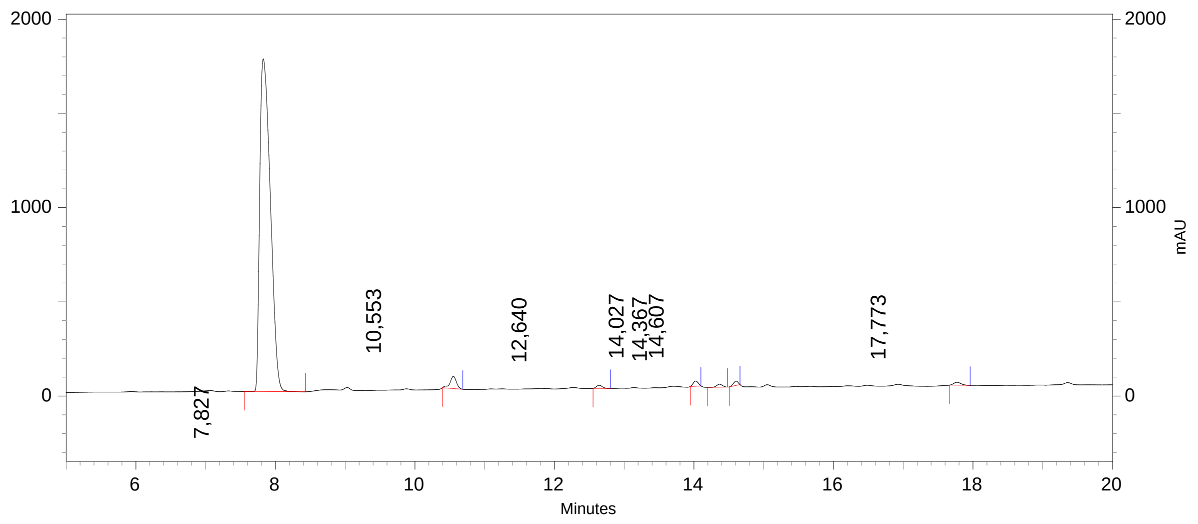

1: 217 nm, 4 nm Results

Retention Time	Area	Area %
7,827	74724482	95,11
10,553	1677072	2,13
12,640	388328	0,49
14,027	546066	0,70
14,367	359156	0,46
14,607	428523	0,55
17,773	442663	0,56
Totals	78566290	100,00

II-*threo*

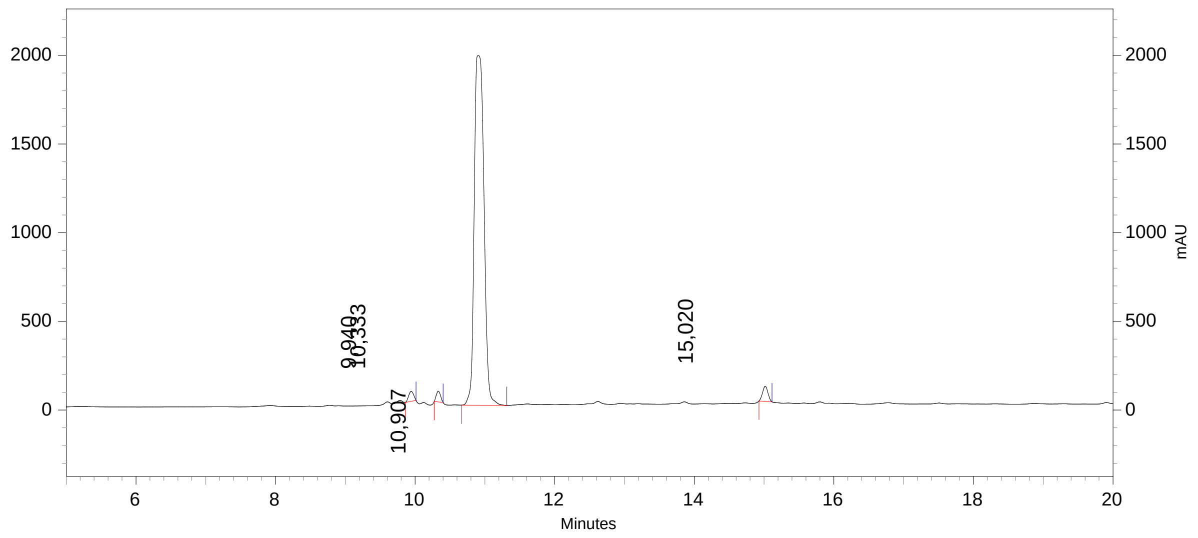

1: 217 nm, 4 nm Results

Retention Time	Area	Area %
9,940	1109636	1,38
10,333	1036785	1,29
10,907	76376532	95,06
15,020	1821479	2,27
Totals	80344432	100,00

III-*threo*

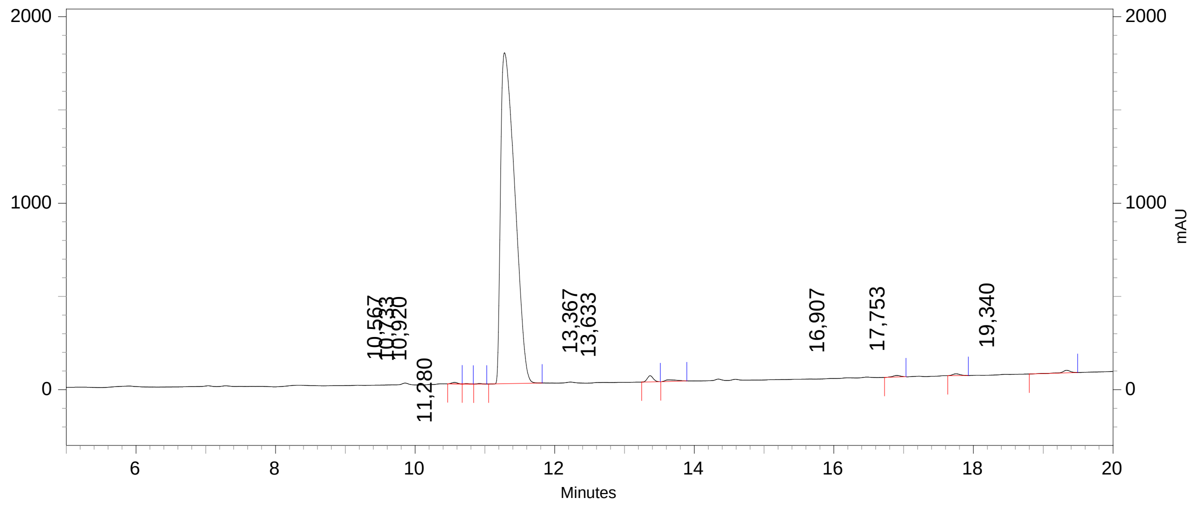

1: 221 nm, 4 nm Results

Retention Time	Area	Area %
10,567	181677	0,18
10,733	43185	0,04
10,920	56195	0,05
11,280	100366633	97,64
13,367	790050	0,77
13,633	391959	0,38
16,907	260625	0,25
17,753	275881	0,27
19,340	425767	0,41
Totals	102791972	100,00

IV-*threo*

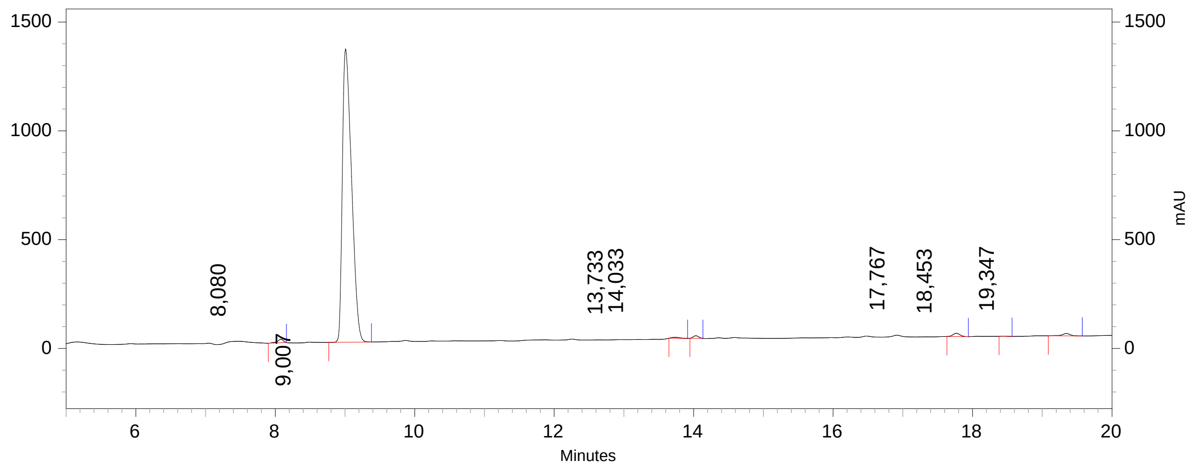

1: 217 nm, 4 nm Results

Retention Time	Area	Area %
8,080	371156	0,73
9,007	49429101	96,85
13,733	146838	0,29
14,033	260262	0,51
17,767	429958	0,84
18,453	20789	0,04
19,347	360918	0,71
21,520	19773	0,04
Totals	51038795	100,00

Table S1:

Calculated binding energy values for MPH and the compounds object of the present paper, obtained from docking studies.

Compound	ΔG (-kcal/mol)
MPH	6.3
I-threo	6.3
I-erythro	6.9
II-threo	8.4
III-threo	7.4
IV-threo	6.7

Table S2:

Radioligand binding assay. Each Ki value represents the arithmetic mean ± S.E.M; n = number of independent experiments, each performed in triplicate. Unpaired t-test with Welch correction was performed to assess difference in binding affinities between I-threo and MPH in DAT. Arithmetic mean and SEM of Ki were utilized from three or four independent experiments. (* P < 0.05).

	**DAT**	
	K_i ± SEM (nM)	n
**I-*threo***	49.1 ± 6.6*	4
**MPH**	117 ± 12.9	3

Table S3:

Literature data on MPH and its analogues on DAT binding and DAT inhibition.

Compound	Compound name in previous reports	$K_i \pm$ SEM (nM)	$IC_{50} \pm$ SEM (nM)
**MPH**	**1**[60] and **1a**[61]	582 ± 77[28]	164 ± 40[26]
**I-*threo***	**1b[61]**	/	114 ± 28[26]
**I-*erythro***	**2b[61]**	/	7430 ± 1520[26]
**II-*threo***	**7e**[62] and **1g**[61]	11.0 ± 2.5[27]	33.9 ± 6.4[26]
**III-*threo***	**1e**[61]	/	1020 ± 110[26]
**IV-*threo***	**44**[26]	/	140 ± 9.0[29]

Material and Methods Binding assay

Radioligand binding assay was performed as previously reported. [64,65] Briefly, for *DAT binding assay*, frozen striatum tissue was dissected from male Sprague Dawley rat brains (supplied on ice by BioIVT, Hicksville, NY) were homogenized in 20 volumes (w/v) of ice-cold modified sucrose phosphate buffer (0.32 M sucrose, 7.74 mM Na_2HPO_4, and 2.26 mM NaH_2PO_4, pH adjusted to 7.4) using a Brinkman Polytron (Setting 6 for 20 s) and centrifuged at 45,995g for 10 min, at 4 °C. The resulting pellet was resuspended in buffer, recentrifuged, and suspended in ice cold buffer again to a concentration of 20 mg/mL, OWW. Experiments were conducted in 96-well polypropylene plates containing 50 µL of various concentrations of the test compound, diluted using 30% DMSO vehicle, 300 µL of sucrose phosphate buffer, 50 µL of [^{3}H]WIN35,428 (final concentration 1.5 nM; *K*d = 14.6 nM; PerkinElmer Life Sciences, Waltham, MA), and 100 µL of tissue (2.0 mg/well OWW). All compound dilutions were tested in triplicate, and the competition reactions started with the addition of tissue; the plates were incubated for 120 min, at 0−4 °C. Nonspecific binding was determined using 10 µM final concentration of indatraline. For all binding assays, incubations were terminated by rapid filtration through PerkinElmer Uni-Filter-96 GF/B $_{\text{(DAT),}}$ presoaked in 0.05% polyethylenimine, using a Brandel 96-Well Plates Harvester manifold or Brandel R48 filtering manifold (Brandel Instruments, Gaithersburg, MD). The filters were washed a total of three times with 3 mL (3 × 1 mL/well or 3 × 1 mL/tube) of ice-cold binding buffer. An amount of 65 µL PerkinElmer MicroScint20 Scintillation Cocktail was added to each filter well. All the plates/filters were counted using a PerkinElmer MicroBeta Microplate Counter. For each experiment, aliquots of the prepared radioligand solutions were

measured to calculate the exact amount of radioactivity added, taking into account the experimentally determined top-counter efficiency for each radioligand. When a complete inhibition could not be achieved at the highest tested concentrations, K_i values have been extrapolated by constraining the bottom of the dose–response curves (= 0% residual specific binding) in the nonlinear regression analysis. K_i values were calculated using GraphPad Prism 8 version 8.4.0 for Macintosh (GraphPad Software, San Diego, CA) utilizing One site—Fit Ki model. K_d values for the radioligands were determined via separate homologous competitive binding or radioligand binding saturation experiments. K_i values were determined from at least three independent experiments performed in triplicate and are reported as mean ± SEM. Statistical differences between l-*threo* and MPH DAT binding were estimated by using unpaired t-test with Welch correction. Arithmetic mean and SEM of K_i were utilized from three or four independent experiments.

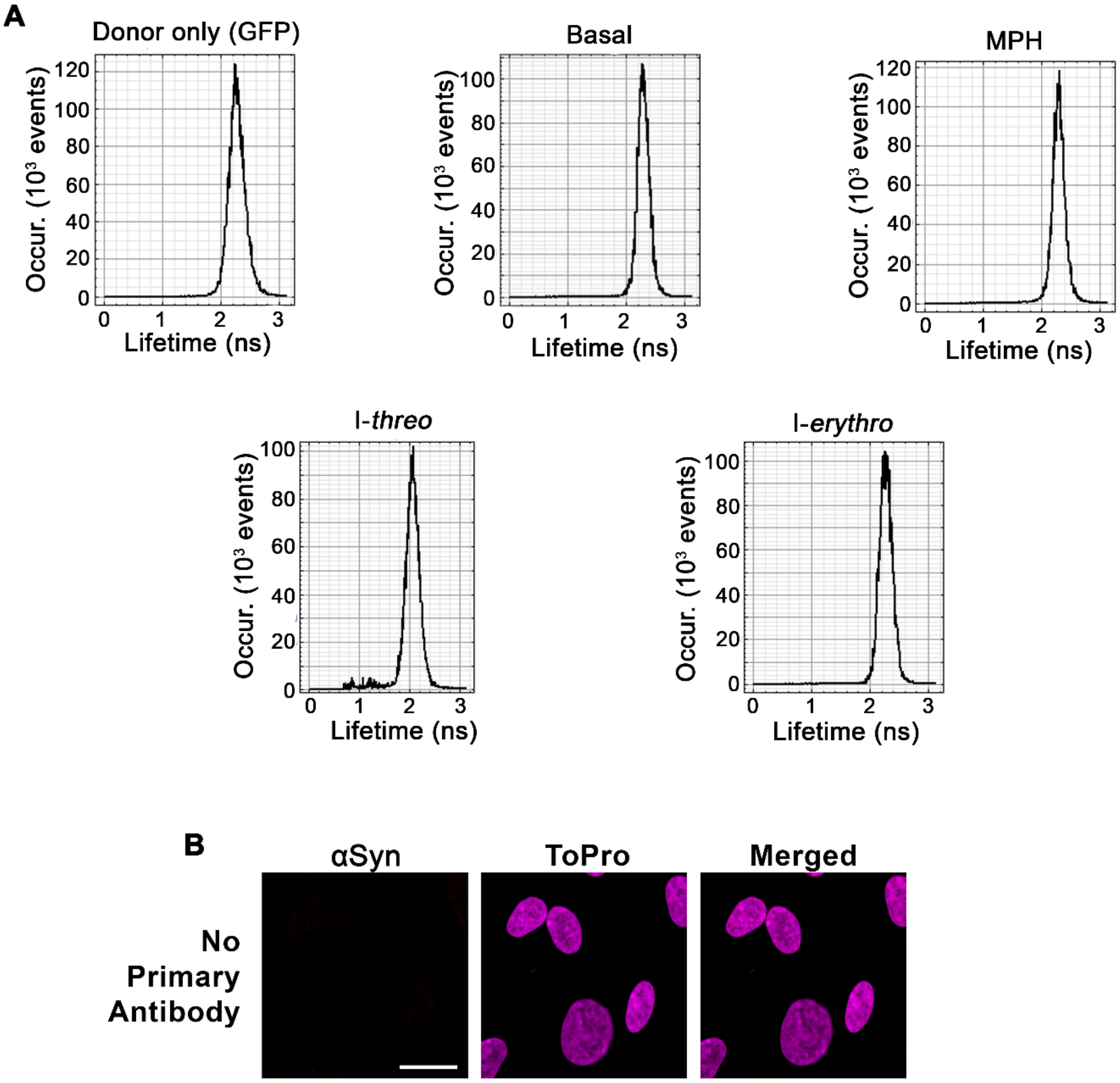

Figure S1. (A) Representative plots showing the lifetime of the single events (GFP photons) in the different conditions, including the negative control (Donor only) performed without the acceptor RFP. **(B)** Images are showing αSyn immunostaining negative control performed on SK-N-SH cell stably overexpressing αSyn by skipping the incubation with the primary antibody (Scale bar=10µm).

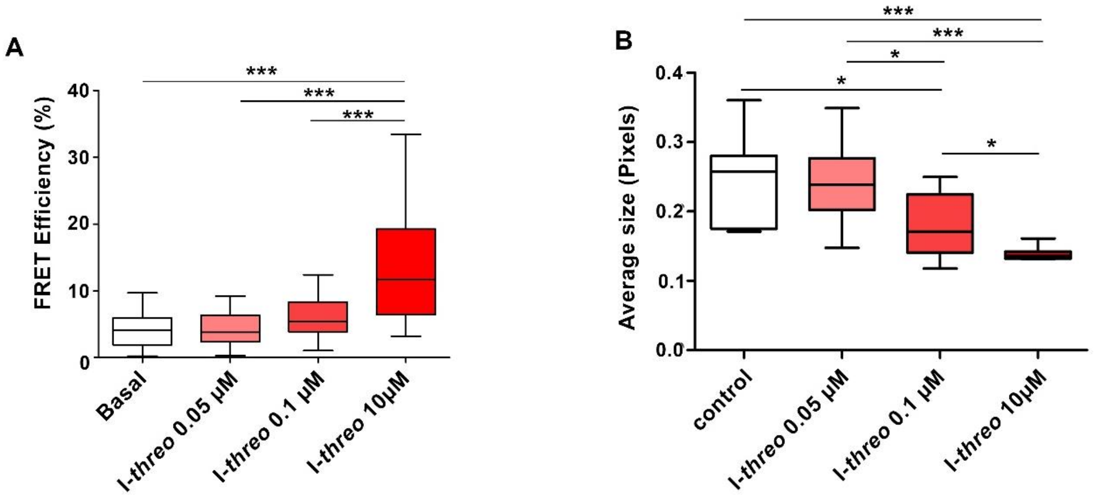

Figure S2. (A) Graph showing FRET efficiency between the GFP/RFP fluorophore couple in SK-N-SH cells treated with **I-*threo*** 0.05, 0.1 and 10 µM for 15 min. Please note that only 10 µM I-threo treatment was able to significantly improve αSyn/Syn III interaction vs. basal condition (*** P<0.001 vs. Basal, 0.05 and 0.1 µM I-threo; One-Way ANOVA with Dunnett's Multiple Comparison Test). **(B)** The graph shows the quantification of the average size of αSyn-positive particles of SK-N-SH cells overexpressing human wt αSyn in basal condition (control) or after treatment with **I-*threo*** 0.05, 0.1 and 10 µM. A significant decrease in the size of αSyn immunopositive dots was observed in the cells treated with 10 µM (*** P<0.001 vs. control, or 0.05 µM **I-*threo***; * P<0.05 vs. 0.1 µM **I-*threo***) and 0.1 µM **I-*threo*** (* P<0.01; One-Way ANOVA with Newman–Keuls Multiple Comparison Test). The treatment with 0.05 µM **I-*threo*** did not decrease the average size of αSyn-positive particle when compared to control condition.

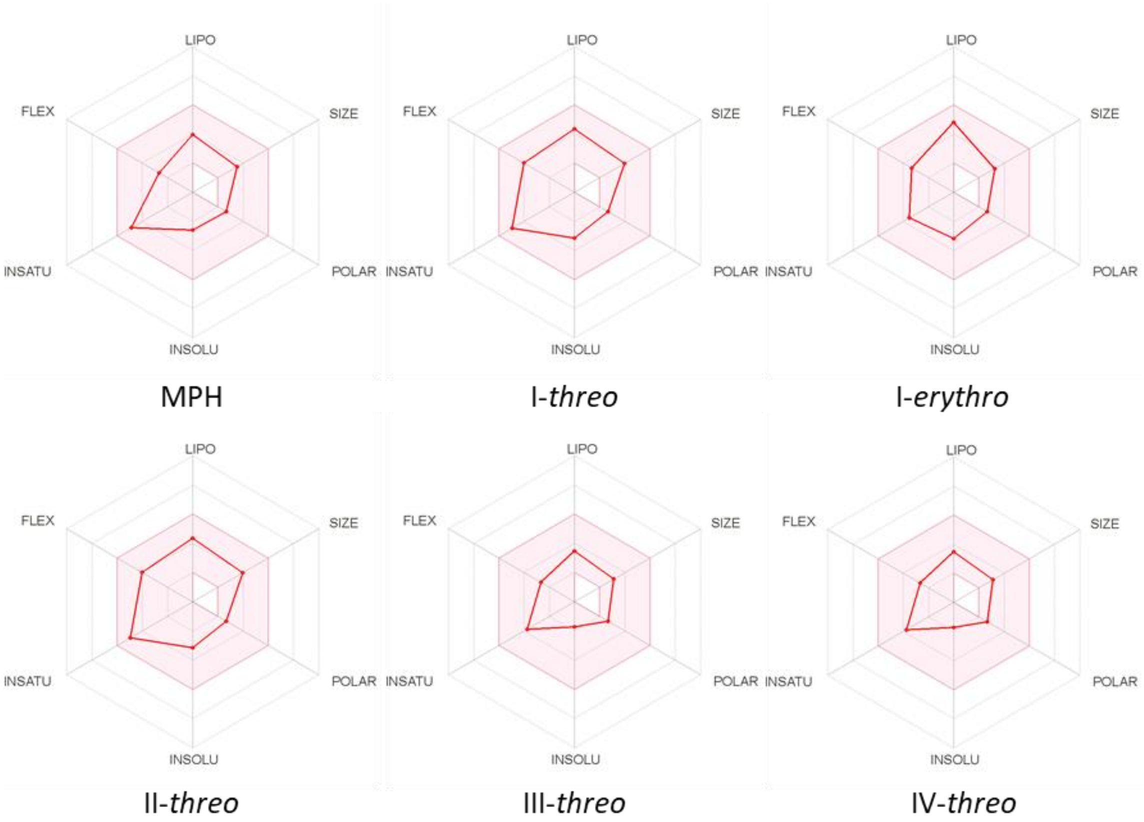

Figure S3. Overview of the predicted physicochemical descriptors for the studied compounds. The red area in the radar graphs represents the suitable physicochemical space for oral bioavailability, according to lipophilicity, size, polarity, solubility, insaturation and flexibility scores of the molecule.

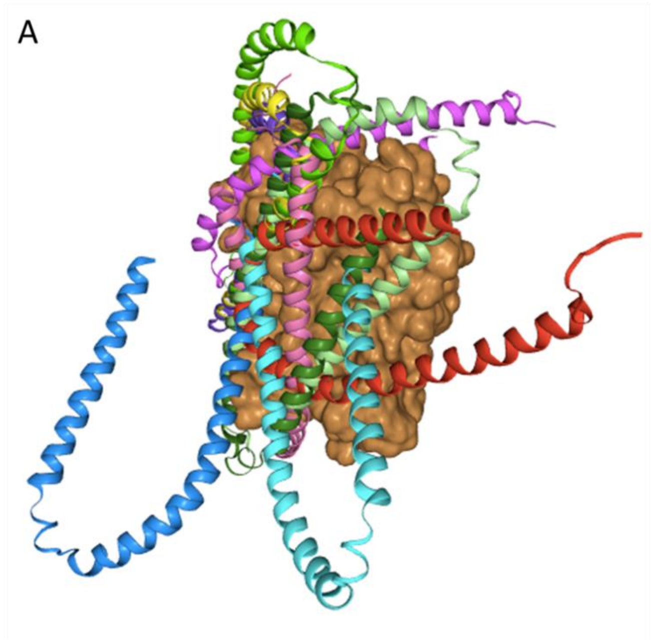

Figure S4. Results generated from the HDOCK protein–protein docking experiment. Synapsin III is depicted in dark orange while the poses of a-synuclein are represented in different colors (A); results of the DeepSite analysis carried out on the complex: orange parts represent the highlighted putative binding sites (B).

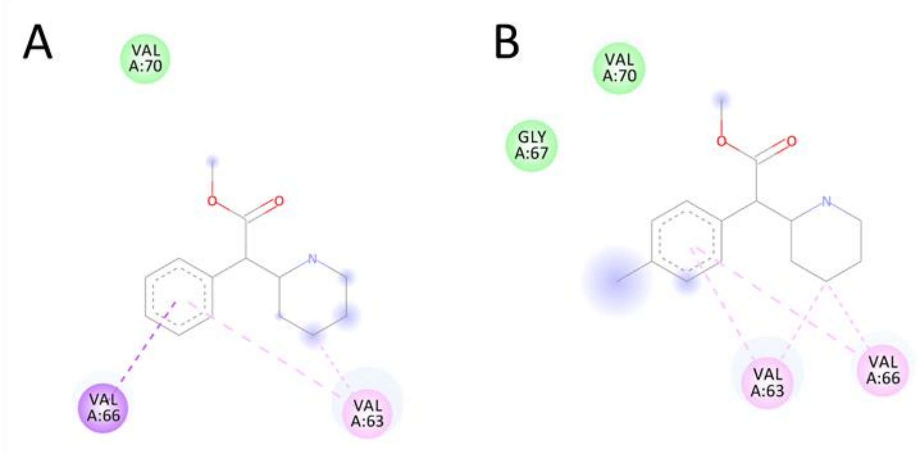

Figure S5. Detailed view of the residues interacting with MPH (A) and l-*threo* (B) in the docked models. This artwork was produced with Discovery Studio software (Dassault Systèmes BIOVIA, San Diego, CA, USA).

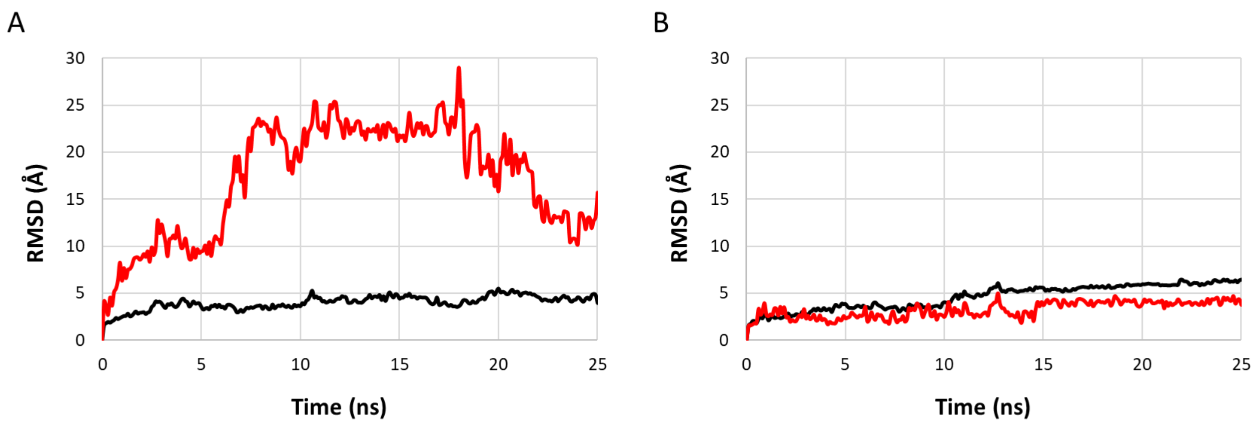

Figure S6. RMSD trajectories obtained from MD simulations performed on the ligand-target complexes generated by docking studies: RMSD variation over time for the MPH-protein complex (A); RMSD variation over time for the I-*threo*-protein complex (B). Protein trajectories are depicted in black, while ligand trajectories are depicted in red.
